# Editorial: Unpacking the Link between Income, Smoking, and Dementia Risk: Insights and Opportunities

**DOI:** 10.31662/jmaj.2025-0231

**Published:** 2025-06-27

**Authors:** Jie V. Zhao

**Affiliations:** 1 Takemi Program in International Health, Harvard T.H. Chan School of Public Health, Boston, USA; 2 School of Public Health, The University of Hong Kong, Hong Kong SAR, China

**Keywords:** smoking, dementia, income

Dementia represents one of the most pressing public health challenges globally, with rising prevalence and substantial impact on society, the economy, and families. Increasing evidence points to the crucial influence of modifiable social determinants, such as socioeconomic position, health behaviors, and environmental exposures. The study by Satomi et al. ^(1)^ offers timely and actionable insights into how income, as a marker of socioeconomic position, relates to dementia risk and how health behaviors, particularly smoking, may mediate this relationship.

The authors present findings from a large, population-based cohort examining the association between income and the risk of dementia, with a particular focus on the mediating role of smoking. The findings suggest that participants with lower income are at a higher risk of dementia. Interestingly, the study uncovers a slightly stronger association in men than in women, although the confidence intervals largely overlap. Importantly, the authors demonstrate that smoking behavior partially mediates the association between low income and dementia risk, contributing more to the mediation than physical inactivity or alcohol use.

This study has both practical and theoretical implications. Income is shaped by broader structural and societal factors and, as such, is not an easy target for direct intervention. However, modifiable lifestyle behaviors like smoking offer feasible and impactful opportunities to curb dementia risk, particularly among socioeconomically disadvantaged populations. Decades of public health efforts have established the link between smoking and diseases such as cardiovascular disease and cancer ^(2)^, conditions that are known contributors to cognitive decline. This study adds dementia to the growing list of conditions that may be linked to smoking.

Despite these strengths, the study warrants careful interpretation within the context of its observational design. Due to the inevitable residual confounding in observational studies, as the authors acknowledged, it can only assess associations, not causality. The possibility of residual confounding, reverse causation (e.g., early cognitive decline influencing health behaviors), and misclassification of exposures or outcomes cannot be fully excluded. Additionally, self-reported lifestyle behaviors such as smoking and alcohol consumption are vulnerable to misclassification. Nonetheless, the study provides some hints on the possible mediation role of smoking.

Beyond its epidemiological contribution, the study opens more compelling questions, such as the biological mechanisms linking smoking to dementia. One hypothesized pathway that has received limited attention to date involves sex hormones. In my previous systematic review and meta-analysis, we found that smoking was associated with elevated testosterone levels in men ^(3)^. The role of testosterone in dementia and cognitive function remains complex and somewhat controversial. Some observational studies have suggested that higher levels of endogenous testosterone may be associated with better cognitive performance in aging men ^(4)^. However, findings from a Mendelian randomization study, which is designed to reduce bias from confounding and reverse causation, have not supported a clear beneficial role for testosterone in improving cognitive function ^(5)^. Future studies exploring the hormonal changes in response to smoking and their cognitive consequences could offer novel insights into dementia pathophysiology, especially in men.

These findings also hold important implications for public health policy. In Japan, where the study was conducted, smoking rates have declined significantly over the past two decades, due in part to legal amendments and public awareness campaigns ^(6)^. However, disparities persist, with higher smoking prevalence among socioeconomically disadvantaged populations ^(1)^. While changing income levels may be complex and slow, intensifying tobacco control, especially among vulnerable groups, offers an immediate opportunity to reduce the cognitive burden associated with health inequality.

In conclusion, this study serves as an important reminder that dementia risk is shaped not only by biology and aging but also by modifiable behaviors and social conditions. The demonstrated role of smoking as a partial mediator between income and dementia risk points to a clear opportunity for intervention. Tobacco control efforts, long championed for cancer and cardiovascular prevention, can now also be viewed as a vital part of dementia prevention, particularly in socioeconomically at-risk groups. Simultaneously, the study opens the door for deeper investigation into biological pathways, such as hormonal changes, that may further influence the smoking-dementia link. By integrating insights from population health, behavioral science, and biology, we can move toward more targeted and inclusive strategies to reduce the burden of dementia in aging societies.

## Article Information

### Conflicts of Interest

None
